# Kv3.4 is modulated by HIF-1α to protect SH-SY5Y cells against oxidative stress-induced neural cell death

**DOI:** 10.1038/s41598-017-02129-w

**Published:** 2017-05-18

**Authors:** Min Seok Song, Pan Dong Ryu, So Yeong Lee

**Affiliations:** Laboratory of Veterinary Pharmacology, College of Veterinary Medicine and Research Institute for Veterinary Science, Seoul National University, Seoul, 08826 Korea

## Abstract

The Kv3.4 channel is characterized by fast inactivation and sensitivity to oxidation. However, the physiological role of Kv3.4 as an oxidation-sensitive channel has yet to be investigated. Here, we demonstrate that Kv3.4 plays a pivotal role in oxidative stress-related neural cell damage as an oxidation-sensitive channel and that HIF-1α down-regulates Kv3.4 function, providing neuroprotection. MPP^+^ and CoCl_2_ are reactive oxygen species (ROS)-generating reagents that induce oxidative stress. However, only CoCl_2_ decreases the expression and function of Kv3.4. HIF-1α, which accumulates in response to CoCl_2_ treatment, is a key factor in Kv3.4 regulation. In particular, mitochondrial Kv3.4 was more sensitive to CoCl_2_. Blocking Kv3.4 function using BDS-II, a Kv3.4-specific inhibitor, protected SH-SY5Y cells against MPP^+^-induced neural cell death. Kv3.4 inhibition blocked MPP^+^-induced cytochrome c release from the mitochondrial intermembrane space to the cytosol and mitochondrial membrane potential depolarization, which are characteristic features of apoptosis. Our results highlight Kv3.4 as a possible new therapeutic paradigm for oxidative stress-related diseases, including Parkinson’s disease.

## Introduction

Voltage-gated potassium (Kv) channels are transmembrane channels that are specific to potassium and sensitive to voltage changes in numerous cells. In neuronal cells, Kv currents play important roles in regulating numerous neurophysiological functions, including resting membrane potential, spontaneous firing rate, and apoptosis, because Kv currents are key regulators of neuronal membrane excitability^1–3^. Shaw-related subfamily (Kv3.1–Kv3.4) Kv channels display rapid activation and deactivation kinetics, as well as relatively large conductance^4^. Among the Kv3 subfamily, Kv3.3 and Kv3.4 are oxygen-sensitive channels, which are also known as oxidation-sensitive channels. Both channels are characterized by fast voltage-dependent inactivation; the cytoplasmic N-terminus has a positively charged ball that provokes the fast closing of the channel by occluding the pore once it is opened^5^. Oxidation of a cysteine residue in the amino terminus of the channels interrupts their fast inactivation by forming a disulfide bond and consequently increasing current amplitude; Kv3.3 and Kv3.4 lose their fast inactivation upon the external application of H_2_O_2_^5, 6^. In the rabbit carotid body, Kv3.4 participates in the chronic hypoxia sensitization of carotid body chemoreceptor cells as an oxygen-sensitive channel; Kv3.4 expression is down-regulated and Kv3.4 current is diminished under hypoxic conditions^7^.

The SH-SY5Y cell line is a thrice cloned subline of SK-N-SH cells, which were established from a neuroblastoma patient^8^. The SH-SY5Y cell line has recently been widely used as an *in vitro* Parkinson’s disease model because SH-SY5Y cells express dopamine transporter (DAT), a dopaminergic neuron-specific protein within the central nervous system. 1-Methyl-4-phenylpyridinium ion (MPP^+^), which is metabolized from 1-methyl-4-phenyl-1,2,3,6-tetrahydropyridine (MPTP) by monoamine oxidase-B (MAO-B), is a neurotoxin that selectively destroys certain dopaminergic neurons in the substantia nigra by interfering with oxidative phosphorylation in mitochondria, thereby depleting ATP and inducing cell death^9, 10^. MPP^+^ requires dopamine transporters for neuronal uptake; therefore, SH-SY5Y cells have been widely utilized as a good model for studying MPP^+^-induced neurotoxicity and the pathogenesis of MPP^+^-induced Parkinson’s symptoms^10^. MPP^+^ is an oxidative stress inducer, and studies suggest that oxidative stress generated by Parkinson’s symptom-inducing reagents such as MPP^+^ and rotenone contribute to their toxicity in SH-SY5Y cells; oxidative stress and free radical generation may play pivotal roles in neurodegeneration^11^. CoCl_2_ is another often-used oxidative stress inducer in SH-SY5Y cells. However, unlike MPP^+^ or rotenone, cobalt stimulates reactive oxygen species (ROS) generation through a non-enzymatic, non-mitochondrial mechanism and CoCl_2_ treatment induces hypoxia-inducible factor 1α (HIF-1α) accumulation^12^. Because HIF-1α accumulates during CoCl_2_ treatment, CoCl_2_ is used as a hypoxia-mimetic agent to investigate the function of HIF-1α.

Kv3.4 is well documented as a potential therapeutic target for Alzheimer’s disease. Kv3.4 is overexpressed in both the early and advanced stages of this neurodegenerative disease, and the up-regulation of Kv3.4 leads to altered electrical and synaptic activity that may underlie the neurodegeneration observed in Alzheimer’s disease^13^. Kv3.4 and its accessory protein MinK-Related Peptide 2 (MIRP2) are involved in neuronal cell death induced by neurotoxic amyloid β-peptide, which is generated from amyloid precursor protein and whose amyloid fibrillar form is the primary component of amyloid plaques found in the brains of Alzheimer’s disease patients^14^. The oxidation-sensitive channel Kv3.4 likely plays a pivotal role in neuronal cell death induced by oxidative stress because oxidative stress is generated from amyloid β-peptide-associated ROS. Furthermore, oxidative stress is one of the general premonitory symptoms of neurodegenerative diseases^15^.

Taken together, oxidative stress is one of the key factors in neurodegenerative diseases such as Alzheimer’s and Parkinson’s disease, and Kv3.4 may be involved in oxidative stress-related abnormal neural cell death as an oxidation-sensitive channel.

## Results

### Kv3.4 mRNA and protein expression levels during CoCl_2_ or MPP^+^ treatment

**Figure 1:**
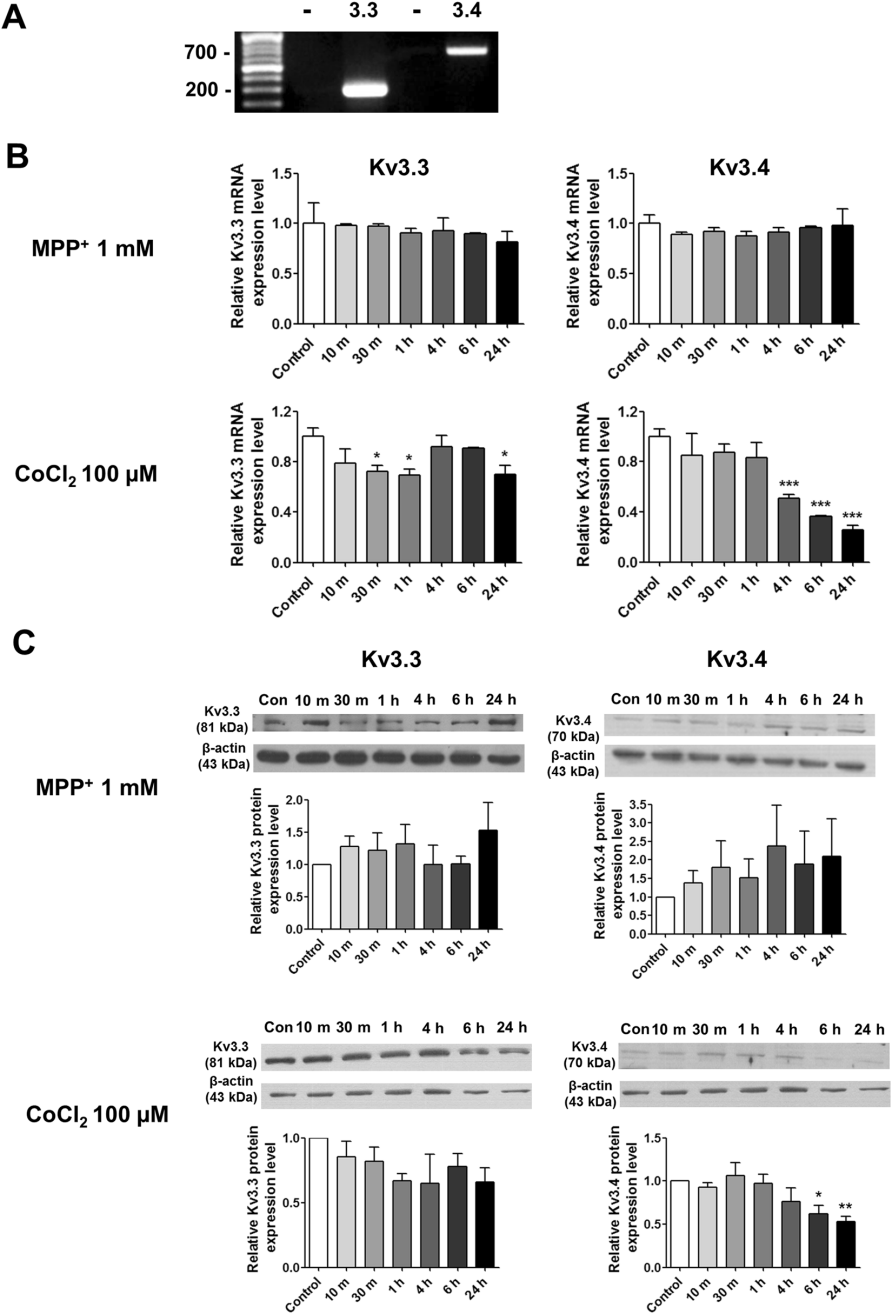
Effects of MPP^+^ and CoCl_2_ on Kv3.3 and Kv3.4 expression in SH-SY5Y cells (**A**) RT-PCR analysis demonstrated that Kv3.3 and Kv3.4 are expressed in SH-SY5Y cells. (**B**) Changes in Kv3.3 and Kv3.4 mRNA expression were measured via qPCR following treatment with 1 mM MPP^+^ or 100 µM CoCl_2_. Neither Kv3.3 nor Kv3.4 were affected by MPP^+^ treatment, whereas Kv3.3 was significantly decreased after 30 min, 1 h, and 24 h of CoCl_2_ treatment. Kv3.4 was decreased after 4, 6, and 24 h of CoCl_2_ treatment. (**C**) Western blotting demonstrated that 1 mM MPP^+^ did not affect Kv3.3 and Kv3.4 protein expression levels. Kv3.3 protein expression was not significantly altered by 100 µM CoCl_2_ treatment, whereas Kv3.4 protein (70 kDa) expression was significantly decreased after 6 and 24 h of 100 µM CoCl_2_ treatment. All experiments were performed in quadruplicate, and data represent the mean ± standard error. *p < 0.05, **p < 0.01 and ***p < 0.001 versus the control value.

RT-PCR analysis reveals that Kv3.3 and Kv3.4 are expressed in SH-SY5Y cells (Fig. 1A). Kv3.3 and Kv3.4 mRNA and protein expression levels were measured at the indicated time-points during MPP^+^ or CoCl_2_ treatment. Kv3.3 and Kv3.4 mRNA expression levels were decreased after 100 µM CoCl_2_ treatment, whereas no change was observed after 1 mM MPP^+^ treatment (Fig. 1B). Kv3.3 mRNA expression was decreased at 30 min, 1 h, and 24 h of CoCl_2_ treatment, and Kv3.4 expression began to decrease after 4 h of CoCl_2_ treatment, which is the time point that ROS started to be accumulated by CoCl_2_ ﻿(Supplementary Fig. 1). The changes in Kv3.3 and Kv3.4 protein expression levels mirrored the changes in mRNA expression during 100 µM CoCl_2_ treatment, but Kv3.4 levels decreased more significantly during treatment. MPP^+^ did not affect Kv3.3 and Kv3.4 protein expression levels, which is consistent with the mRNA expression data (Fig. 1C).

### Changes in BDS-II sensitive currents during CoCl_2_ or MPP^+^ treatment

**Figure 2:**
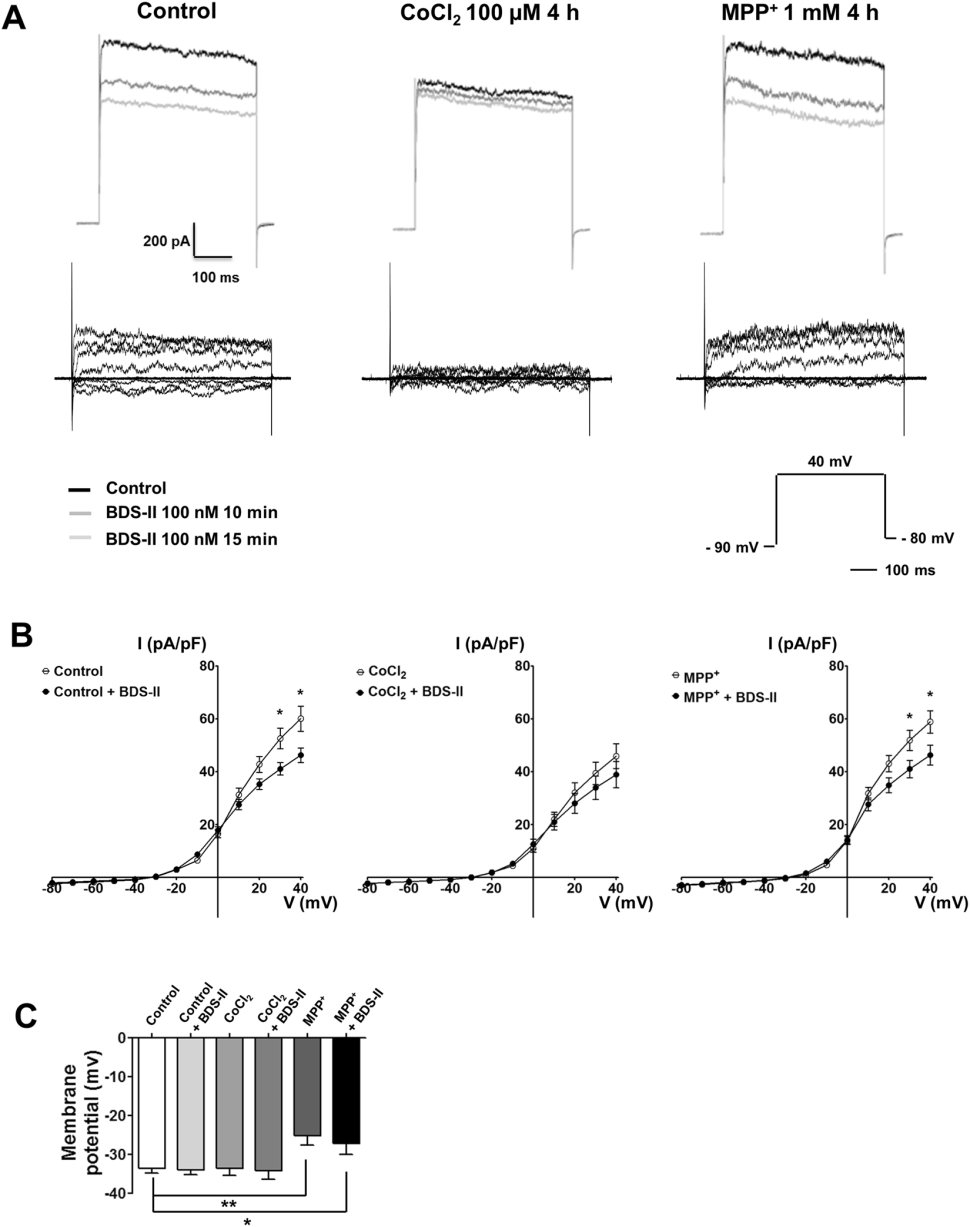
Patch-clamp recordings of BDS-II-sensitive currents during CoCl_2_ or MPP^+^ treatment (**A**) Representative whole-cell currents recorded in SH-SY5Y cells. Currents sensitive to BDS-II (100 nM) were measured in whole-cell mode. SH-SY5Y cells were hyperpolarized by a −90 mV pulse and then depolarized by a + 40 mV pulse with a −80 mV holding potential. After 10 min of BDS-II treatment, diminished currents were observed in the control group (327.62 ± 83.45 pA, n = 12), the 100 µM CoCl_2_-treatment group (138.89 ± 51.8 pA, n = 8), and the 1 mM MPP^+^-treatment group (253.3 ± 47.98 pA, n = 9). (**B**) BDS-II significantly inhibited voltage-dependent currents at +30 and +40 mV of depolarization in the control group and 1 mM MPP^+^-treatment group, whereas no BDS-II-sensitive current was detected in cells treated with 100 µM CoCl_2_. Experiments were repeated at the indicated times, and data are expressed as the mean ± standard error. *p < 0.05 and ***p < 0.001 versus the control value. (**C**) The resting membrane potential of SH-SY5Y cells was significantly depolarized by 1 mM MPP^+^ treatment. However, 100 nM BDS-II did not affect the resting membrane potential.

Kv3.4-related currents were investigated in SH-SY5Y cells using patch-clamp recording. Currents sensitive to BDS-II, a Kv3.4-selective channel blocker, were reduced in cells treated with 100 µM CoCl_2_, whereas 1 mM MPP^+^ did not affect BDS-II-sensitive currents (Fig. 2A). BDS-II-sensitive currents were detected when we applied a greater than +30 mV pulse to the control group and the MPP^+^-treatment group (Fig. 2B). However, in the CoCl_2_ treatment group, only a small number of BDS-II-sensitive currents were detected in SH-SY5Y cells (Fig. 2B). BDS-II-sensitive currents were analysed after applying a + 40 mV pulse to the control group (327.62 ± 83.45 pA, n = 12), CoCl_2_ treatment group (138.89 ± 51.8 pA, n = 8), and MPP^+^ treatment group (253.3 ± 47.98 pA, n = 9) (Fig. 2B). The resting membrane potential was depolarized by 1 mM MPP^+^ treatment. However, 100 nM BDS-II treatment had no effect on the resting membrane potential (Fig. 2C).

### HIF-1α regulates Kv3.4 expression levels in SH-SY5Y cells

**Figure 3:**
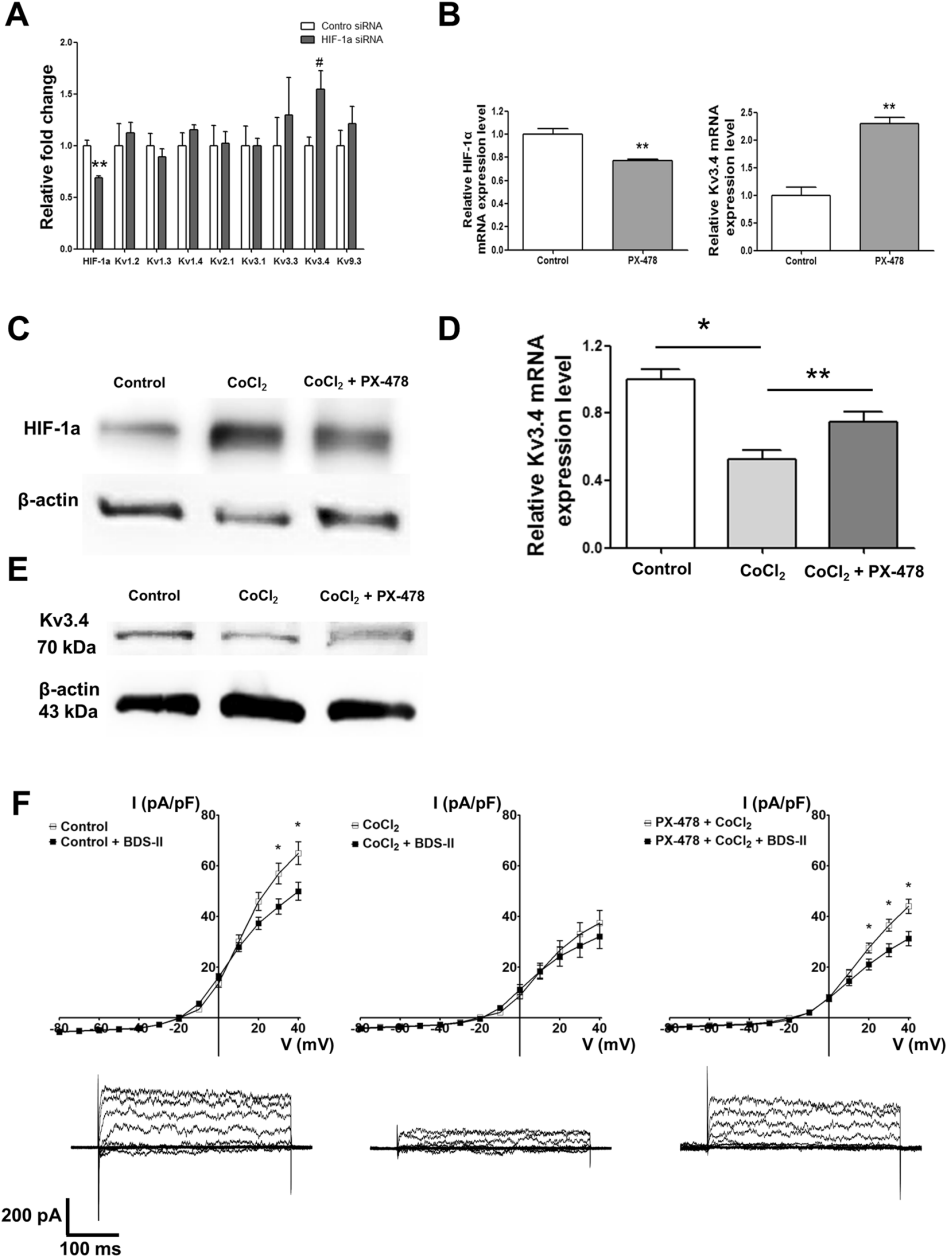
The relationship between HIF-1α and Kv3.4 (**A**) Out of eight oxygen-sensitive Kv channels (Kv1.2, Kv1.3, Kv1.4, Kv2.1, Kv3.1, Kv3.3, Kv3.4, and Kv9.3), only Kv3.4 from among eight was significantly up-regulated by transient HIF-1α siRNA transfection. (**B**) PX-478 (40 µM), a HIF-1α-specific blocker, inhibited HIF-1α and up-regulated Kv3.4 mRNA expression. (**C**) HIF-1α accumulated upon 100 µM CoCl_2_ treatment, and CoCl_2_-induced HIF-1α accumulation was inhibited by 40 µM PX-478 treatment: 4 h of PX-478 pretreatment followed by 6 h of CoCl_2_ and PX-478 treatment. (**D**) Kv3.4 mRNA expression was down-regulated after 4 h of 100 µM CoCl_2_ treatment. PX-478 pretreatment recovered the down-regulation of Kv3.4 mRNA expression induced by CoCl_2_. (**E**) Kv3.4 (70 kDa) was decreased after 6 h of CoCl_2_ treatment. The decrease in Kv3.4 (70 kDa) expression was recovered by PX-478 pretreatment (4 h) followed by 6 h of CoCl_2_ and PX-478 treatment. (**F**) Currents sensitive to BDS-II (100 nM) were detected at +30 and +40 mV of depolarization (n = 6), whereas BDS-II-sensitive currents were diminished by 100 µM CoCl_2_ treatment (n = 6). Diminished BDS-II-sensitive currents were recovered by 40 µM PX-478 pretreatment at +20, +30, and +40 mV of depolarization (n = 9). Experiments were repeated in triplicate or the indicated times, and data represent the mean ± standard error. *, ^#^p < 0.05 and **p < 0.01 versus the control value.

Because HIF-1α accumulates in response to CoCl_2_ but not MPP^+^ treatment, HIF-1α is a strong candidate for regulating Kv3.4 expression in SH-SY5Y cells. Kv3.4 was most significantly affected by siRNA among Kv channels (Fig. 3A). Treating cells with PX-478, a HIF-1α-selective inhibitor^16^, led to the down-regulation of HIF-1α and also affected Kv3.4 expression levels by up-regulating Kv3.4 mRNA expression (Fig. 3B,C). Next, we investigated whether PX-478 inhibited the changes Kv3.4 expression during CoCl_2_ treatment. PX-478 rescued the down-regulation of Kv3.4 mRNA expression caused by CoCl_2_ (Fig. 3D). Kv3.4 protein expression, which was down-regulated after 6 h of CoCl_2_ treatment, was also recovered when we pretreated with PX-478 for 4 h and treated with CoCl_2_ with PX-478 for 6 h (Fig. 3E). In addition, PX-478 also recovered the reduction in BDS-II-sensitive Kv currents induced by CoCl_2_ treatment (Fig. 3F).

### Mitochondrial Kv3.4 is important in oxidative stress regulation

**Figure 4:**
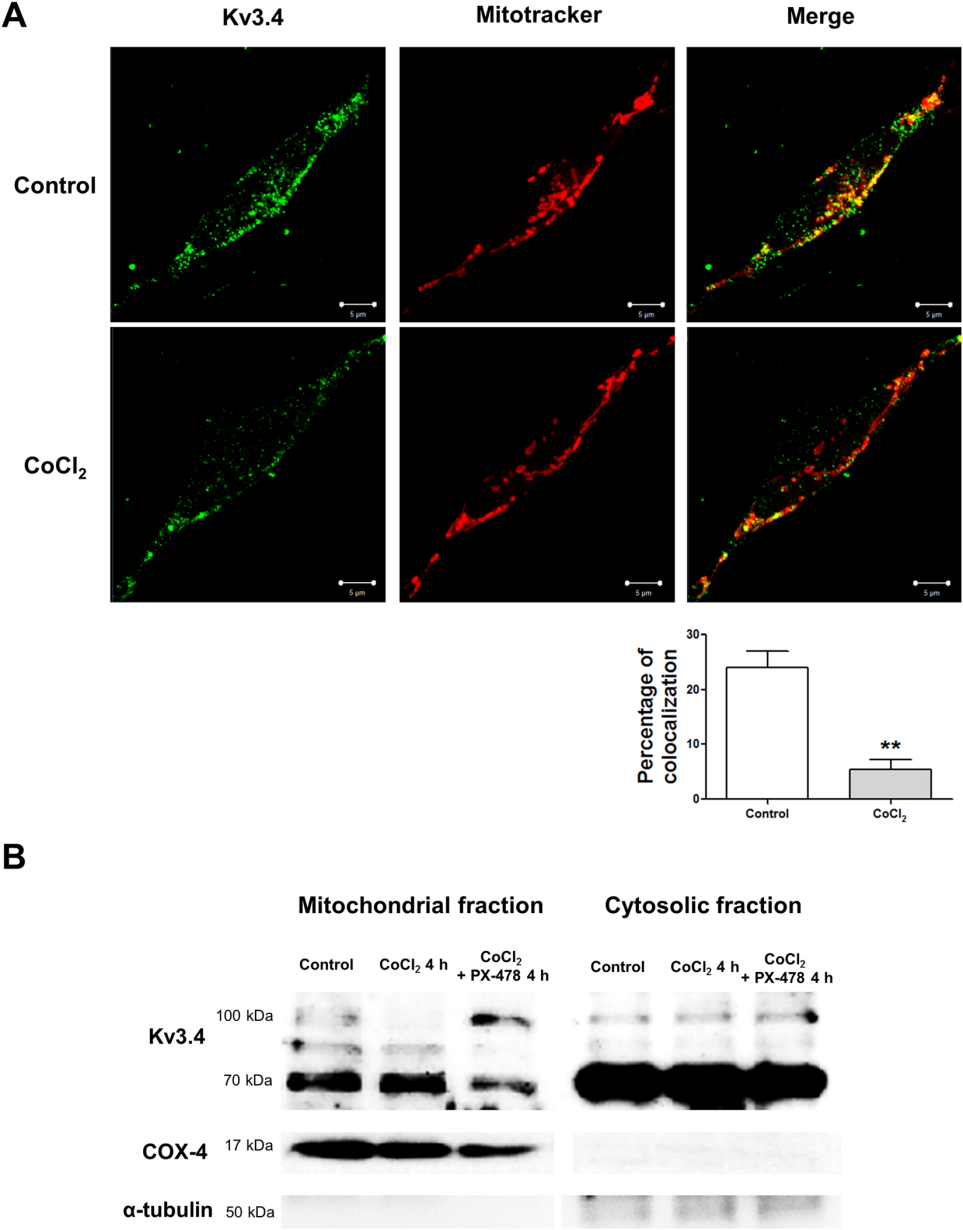
Mitochondrial Kv3.4 was more sensitive to CoCl_2_ and PX-478 (**A**) Immunocytochemical analysis demonstrated that Kv3.4 co-localizes with mitochondria, and co-localized Kv3.4 decreased after 6 h of 100 µM CoCl_2_ treatment (green: Kv3.4, red: mitochondria, yellow: co-localization). We assessed five different images in each group (the control and CoCl_2_ treatment groups) to avoid cherry-picking; representative images are shown. (**B**) Mitochondrial fractionation demonstrated that Kv3.4 is present in both the mitochondria and cytosol and that mitochondrial 100 kDa Kv3.4 is sensitive to CoCl_2_ alone or CoCl_2_ with PX-478 treatment. Kv3.4 (100 kDa) was down-regulated by 4 h of CoCl_2_ treatment, whereas 4 h of PX-478 pretreatment followed by 4 h of CoCl_2_ and PX-478 treatment induced the accumulation of Kv3.4. COX-4 and α-tubulin are used as mitochondrial and cytosolic markers, respectively. All experiments were performed in quadruplicate; representative images are shown.

Mitochondrial potassium channels protect neuronal tissues, and their putative functional roles include not only physiological mitochondrial properties but also the modulation of reactive oxygen species generation in mitochondria^17^. Therefore, we examined whether Kv3.4 exists in mitochondria. Immunocytochemical analysis demonstrated that Kv3.4 (green) and mitochondria (red) are co-localized and co-localization of Kv3.4 was decreased after 6 hours of CoCl_2_ treatment (Fig. 4A). We then explored why Kv3.4 protein expression was unaltered despite the down-regulation of Kv3.4 mRNA expression after 4 h of CoCl_2_ treatment (Fig. 1B,C). Figure 4B demonstrates that cytosolic Kv3.4 protein expression is not affected by 4 h of CoCl_2_ treatment, whereas mitochondrial Kv3.4 is affected by the same experimental condition. Western blotting demonstrated that the 100 kDa Kv3.4 band was more dramatically altered compared with the 70 kDa Kv3.4 band in cells treated with CoCl_2_ or CoCl_2_ plus PX-478. The 100 kDa Kv3.4 band accumulated upon PX-478 treatment (Fig. 4B).

### HIF-1α affects the NDFIP1-related Kv3.4 regulatory system

**Figure 5:**
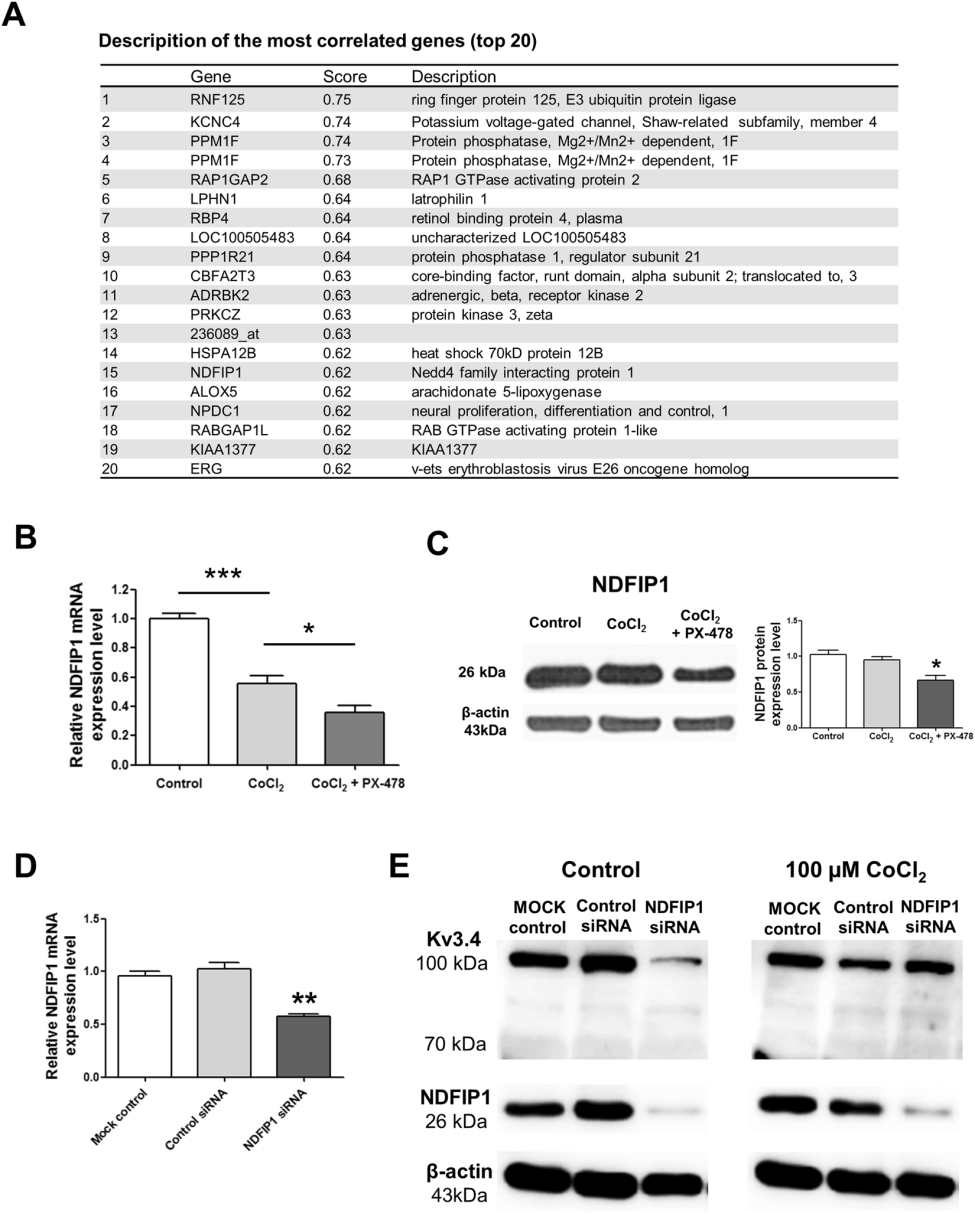
HIF-1α and NDFIP1-related Kv3.4 regulatory system (**A**) The co-expression analysis tool in GENEVESTIGATOR^®^ was used to predict genes that are co-expressed with Kv3.4 during apoptosis. The genes were ranked based on their correlation coefficient. Nedd4 family interacting protein 1 (NDFIP1) ranked 15^th^ (0.62 score). (**B**) NDFIP1 mRNA expression was down-regulated by 100 µM CoCl_2_ treatment, and 40 µM PX-478 pretreatment additionally down-regulated NDFIP1 mRNA expression. (**C**) CoCl_2_ (100 µM) did not affect NDFIP1 protein expression, whereas 40 µM PX-478 pretreatment followed by CoCl_2_ and PX-478 treatment down-regulated NDFIP1 protein expression. (**D**) NDFIP1 siRNA efficiently down-regulated NDFIP1 mRNA expression by half. (**E**) NDFIP1 siRNA down-regulated NDFIP1 protein expression and 100 kDa Kv3.4 expression. When cells were treated with 100 µM CoCl_2_, NDFIP1 siRNA did not affect 100 kDa Kv3.4 protein expression, despite the efficient down-regulation of NDFIP1 protein levels by NDFIP1 siRNA. All of the experiments were performed in triplicate, and data are expressed as the mean ± standard error. *p < 0.05, ***p < 0.001 versus the control value.

We found that ubiquitination is closely related to Kv3.4 when cells enter apoptosis using GENEVESTIGATOR®, a bioinformatic tool. Ring finger protein 125, E3 ubiquitin protein ligase (RNF125), and Nedd4 family-interacting protein 1 (NDFIP1) are highly co-expressed with Kv3.4 (Fig. 5A). According to previous reports, NDFIP1 protein expression was up-regulated when SH-SY5Y cells were treated with CoCl_2_ at concentrations above 200 µM, whereas 100 µM CoCl_2_ did not affect NDFIP1^18, 19^. In addition, 100 µM CoCl_2_ did not up-regulate NDFIP1 protein expression, and NDFIP1 mRNA levels were down-regulated by 100 µM CoCl_2_ treatment in SH-SY5Y cells (Fig. 5B,C). To confirm the relationship between HIF-1α and NDFIP1, we pretreated cells with PX-478 followed by PX-478 and CoCl_2_ treatments. NDFIP1 mRNA and protein expression levels were down-regulated by CoCl_2_ plus PX-478 (Fig. 5C). We hypothesized that CoCl_2_-induced HIF-1α accumulation up-regulates NDFIP1. In addition, given that PX-478 pretreatment not only inhibits CoCl_2_-induced HIF-1α accumulation but also removes the remaining HIF-1α that originally exists in SH-SY5Y cells, PX-478 treatment down-regulates NDFIP1. Next, we examined the relationship between Kv3.4 and NDFIP1. NDFIP1 siRNA effectively down-regulated the expression of 100 kDa Kv3.4 (Fig. 5D,E). On the other hand, NDFIP1 siRNA did not affect the expression of 100 kDa Kv3.4 in CoCl_2_-treated cells (Fig. 5E).

### The neuroprotective effect of BDS-II against MPP^+^-induced SH-SY5Y cell death

**Figure 6:**
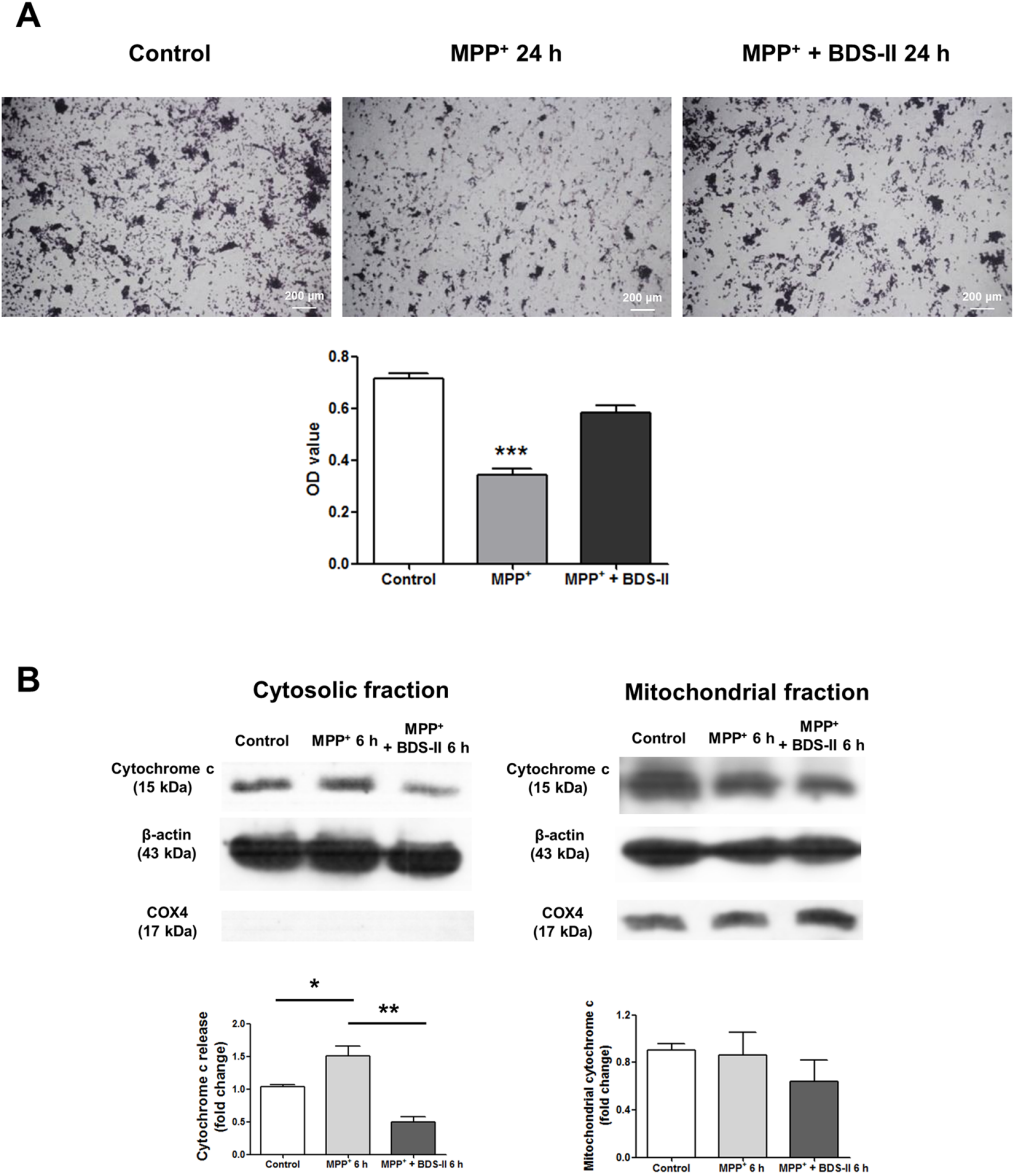
The neuroprotective effect of BDS-II against MPP^+^-induced SH-SY5Y cell death (**A**) Hemacolor-stained SH-SY5Y cells and MTT assays demonstrate that 100 nM BDS-II protected SH-SY5Y cells against 500 µM MPP^+^-induced neural cell death. The MTT assay demonstrated that only 48.23 ± 2.91% of cells survived after 24 h of 500 µM MPP^+^ treatment, whereas BDS-II enabled 81.68 ± 3.94% of cells to survive after 500 µM MPP^+^ treatment. (**B**) BDS-II blocked MPP^+^-induced cytochrome c release from the mitochondrial intermembrane space to the cytosol, which is a key step in the apoptosis signalling pathway. Experiments were repeated in triplicate, and data represent the mean ± standard error. *p < 0.05, **p < 0.01, and ***p < 0.001 versus the control value.

HIF-1α has a neuroprotective effect against rotenone-induced injury, which mimics Parkinson’s disease symptoms^20^. The down-regulation of Kv3.4 by HIF-1α during CoCl_2_ treatment may have a neuroprotective effect on SH-SY5Y cells; thus, we applied this scheme to MPP^+^ treatment. As shown in Fig. 6A, BDS-II pretreatment protected SH-SY5Y cells from MPP^+^-induced cell death. Hemacolor fast staining and MTT assay data demonstrated that 100 nM BDS-II, the lowest effective concentration indicated in the product datasheet, rescued SH-SY5Y cells from 500 µM MPP^+^-induced neural cell death (Fig. 6A). BDS-II treatment blocked MPP^+^-induced cytochrome c release from the mitochondrial intermembrane space to the cytosol, which is one of the key steps of the apoptotic pathway (Fig. 6B).

### BDS-II blocked MPP^+^-induced MMP depolarization in SH-SY5Y cells

**Figure 7:**
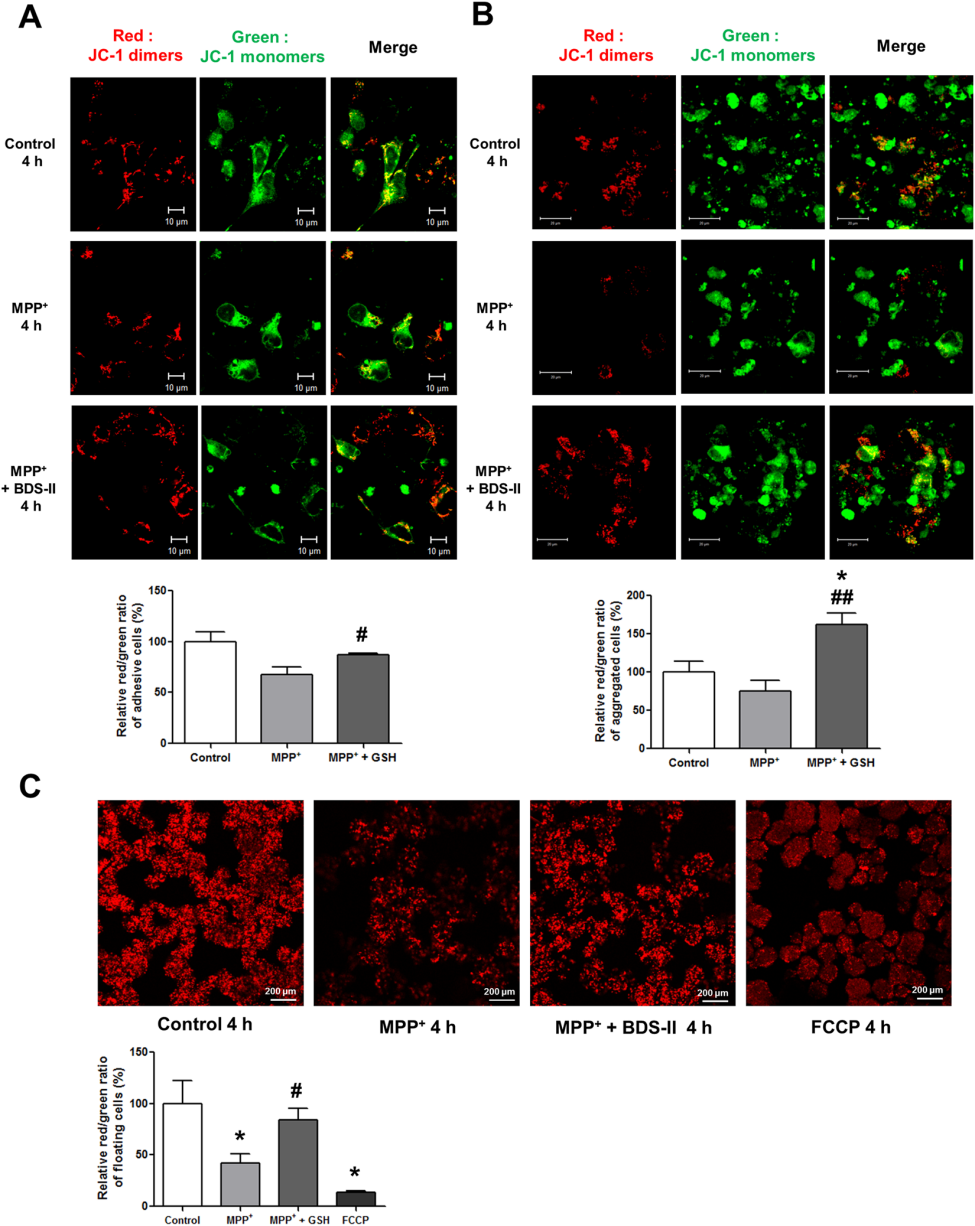
BDS-II blocked MPP^+^-induced MMP depolarization in SH-SY5Y cells (**A**) JC-1 staining revealed that BDS-II prevented the depolarization of mitochondrial membrane potential induced by 1 mM MPP^+^ in SH-SY5Y cells. Red JC-1 dimers represent normal mitochondrial membrane potential. Green JC-1 monomers represent depolarized mitochondrial membrane potential. Red dimers and green monomers were co-localized in control cells. When cells were treated with 1 mM MPP^+^, most of the cells showed only green monomers. When we pretreated with 100 nM BDS-II for 10 min followed by 4 h of 1 mM MPP^+^ and 100 nM BDS-II treatment, red dimers and green monomers co-localized again, but many cells also exclusively exhibited red dimers. (**B**) SH-SY5Y cells can form aggregates of undifferentiated cells. BDS-II also prevented the depolarization of mitochondrial membrane potential induced by 1 mM MPP^+^ in aggregated SH-SY5Y cells. Red dimers and green monomers were detected in the control and MPP^+^ + BDS-II groups, whereas almost all of the red dimers disappeared in the MPP^+^ group. (**C**) SH-SY5Y cells also grew into floating clumps of cells. Red JC-1 dimers indicated that 100 nM BDS-II rescued SH-SY5Y cells from MMP depolarization induced by 1 mM MPP^+^ treatment. FCCP (100 µM) was used as a positive control. All of the experiments were performed in triplicate or quadruplicate, and representative images are shown.

The mitochondrial membrane potential (MMP) was also measured using JC-1 staining. The MMP of control-group cells adhered to the floor of the cell plate indicated that red normal MMP and green depolarized MMP were co-localized, indicating that MMP is regulated by the surrounding microenvironment (Fig. 7A). Following MPP^+^ treatment, green JC-1 monomers increased, indicating that the MMP of the adhered cells was depolarized, whereas BDS-II pretreatment blocked the MPP^+^-induced MMP depolarization (Fig. 7A). Undifferentiated SH-SY5Y cells have the ability to form aggregates; when the cells are differentiated, they spread into the surrounding area^21^. Therefore, we also measured the MMP of aggregated SH-SY5Y cells. MPP^+^ depolarized the MMP of the SH-SY5Y aggregates, and BDS-II pretreatment again blocked depolarization (Fig. 7B). The MMP of the floating SH-SY5Y cells was also measured because SH-SY5Y cells not only grow while adhered to the substrate but also grow in clumps that float in the media. The MMP of the MPP^+^-treated cells was depolarized, whereas the BDS-II pretreatment group exhibited a normal MMP compared with the control group (Fig. 7C). As a control, the MMP-depolarizing agent FCCP depolarized floating cells; no cells remained adhered to the plate when treated with FCCP (Fig. 7C).

## Discussion

Previous studies have revealed the cellular functions of Kv channels, including cell proliferation, apoptosis, and oxygen sensing^5, 22–24^. Kv3.4 is characterized by its fast inactivation, and the channel loses its fast inactivation property upon external application of H_2_O_2_ and this effect is reversible if glutathione is added as a reducing agent; therefore, Kv3.4 is an oxidation-sensitive channel^5, 6^. However, to date, most of the previous studies investigated Kv3.4 exclusively as a hypoxia-related and oxygen-sensitive channel, although there are numerous oxidation-inducing factors other than oxygen^7, 25^.

In the present study, we demonstrated that Kv3.4 may regulate oxidative stress as an oxidation-sensitive channel. First, we treated SH-SY5Y cells with MPP^+^, an *in vitro* Parkinson’s disease-inducing reagent, as a ROS-generating reagent. However, no change in Kv3.4 mRNA or protein expression levels was observed, and patch-clamp recordings also demonstrated that MPP^+^ treatment did not affect BDS-II-sensitive currents. We hypothesized that Kv3.4 is not involved in the regulation of oxidative stress induced by MPP^+^, perhaps because the specific cellular defence mechanism against oxidative stress that includes Kv3.4 is not activated. Next, we examined whether ROS generated by CoCl_2_ may lead to Kv3.4-related oxidative stress regulation, since CoCl_2_ is not only an oxidative stress inducer but also a hypoxia-mimetic reagent and several reports have addressed the relationship between hypoxia and Kv3.4^7, 25, 26^. When we treated the cells with CoCl_2_, Kv3.4 mRNA and protein expression levels were reduced; patch clamp recordings also demonstrated that BDS-II-sensitive currents disappeared after CoCl_2_ treatment. According to our results, although both CoCl_2_ and MPP^+^ induce ROS generation, only CoCl_2_ treatment down-regulates the Kv3.4 expression level and BDS-II-sensitive currents. These results indicate that a key factor for Kv3.4 regulation exists or is activated exclusively by CoCl_2_ treatment, not MPP^+^ treatment.

We hypothesized that HIF-1α is a key factor in the regulation of Kv3.4 because HIF-1α accumulates under CoCl_2_ but not under MPP^+^ treatment^20^. Pretreatment with the HIF-1α-specific inhibitor PX-478 prior to CoCl_2_ treatment restored the CoCl_2_-induced reduction in BDS-II-sensitive currents. Previous studies found that decreased reactive-oxygen intermediates, including H_2_O_2_, caused by a low level of available substrate oxygen during hypoxia resulted in reduced production of oxidized Kv3.4 and that Kv3.4 currents were subsequently reduced^7, 27^. However, Kv3.4 currents were also reduced by CoCl_2_, a well-known ROS inducer^12, 28^, in our experiments. Moreover, hypoxic conditions increase ROS production and oxidative stress^7, 12, 28^. Therefore, the reduced Kv3.4 currents during hypoxia may be regulated by HIF-1α. In fact, partial HIF-1α deficiency in pulmonary artery smooth muscle cells appeared to restore the chronic hypoxia-induced reduction in Kv currents^29^.

Mitochondrial dysfunction has been implicated in several models of acute and chronic neuronal death, and the major chronic neurodegenerative diseases, such as Alzheimer’s, Parkinson’s, or Huntington’s disease, display depolarized MMP and apoptosis^30–32^. Therefore, agents targeting specific mitochondrial ion channels or proteins that contribute to the MMP would be useful therapeutic tools to inhibit acute cell death^32^. In pulmonary artery smooth muscle cells, changes in the localization of mitochondria and mitochondria-dependent intracellular ATP and Mg^2+^ concentrations are closely related to Kv currents^33, 34^. In particular, the existence of mitochondrial Kv1.3 has been substantiated, and mitochondrial Kv1.3 mediates Bax-induced apoptosis in lymphocytes^17, 35, 36^. Immunocytochemistry and mitochondrial fractionation suggest that Kv3.4 is present on mitochondria and plays a role in MMP-related apoptosis in SH-SY5Y cells. Kv3.4 expression levels change during 4-h CoCl_2_ and PX-478 treatment, which were not detected in Western blots of total protein but only in the mitochondrial fraction. Interestingly, 100 kDa Kv3.4 was more dramatically altered than 70 kDa Kv3.4 by CoCl_2_ treatment. Although the general size of Kv3.4 is 70 kDa, the size of Kv3.4 varies because Kv channels form tetramers and have some accessory channels that regulate their currents. Kv3.4 is 100 kDa when it is glycosylated or forms a tetramer with Kv3.1 or other accessory channels, including Mirp2^37–39^. Based on our results, we conclude that such a glycosylated form or channel heteromers, which include accessory channel proteins, aid in the subcellular localization of Kv3.4, including to mitochondria. We also conclude that mitochondrial Kv3.4 is more sensitive to oxidative stress.

Bioinformatic analysis demonstrates that ubiquitination is closely related to Kv3.4 during cell death, and we focused on NDFIP1 because the neural precursor cell expressed developmentally down-regulated protein 4 (Nedd4) family, including Nedd4 and Nedd4-2, which use NDFIP1 as an intermediate adaptor protein, are related to the regulation of voltage-gated ion channels in excitable cells^40^. Nedd4 and Nedd4-2 are E3 ubiquitin ligase enzymes that target proteins for ubiquitination, and NDFIP1 is one of the adaptor proteins required to bring Nedd4 and Nedd4-2 to the target proteins^40^. Our data demonstrate that NDFIP1 siRNA down-regulated Kv3.4 efficiently. However, when the cells were treated with CoCl_2_, NDFIP1 siRNA did not affect Kv3.4 expression. HIF-1α plays a pivotal role in Kv3.4 regulation; therefore, we hypothesize that accumulated HIF-1α potentially affected NDFIP1-related Kv3.4 regulation.

Considering our data, we hypothesized that CoCl_2_ may have an effect on the oxidative stress-regulating systems that are related to Kv3.4, whereas MPP^+^ does not affect Kv3.4. The regulation of Kv3.4 protein expression by HIF-1α, which are accumulated by CoCl_2_, may not be enough to activate the Kv3.4 related oxidative stress-regulating system, however, we believe that it would activate the oxidative stress-regulating system. Because a low CoCl_2_ concentration (50, 100, 150, or 200 μM) shows cytoprotection in cardiomyocytes and HepG2 cells^41, 42^ and Kv3.4 is down-regulated by 100 μM of CoCl_2_ in our data. Therefore we believed down-regulated Kv3.4 is involved in the cytoprotection induced by a low CoCl_2_ concentration. On the other hand, a high CoCl_2_ concentration (500 μM) prompts cell death in the SH-SY5Y cells^43^, and in our scheme, it is because ROS generated by a high concentration of CoCl_2_ may overcome the cytoprotective effect of downregulated Kv3.4. According to our hypothesis, inhibiting Kv3.4 would alter the response of SH-SY5Y cells to MPP^+^ treatment. We therefore treated cells with BDS-II, a selective Kv3.4 inhibitor, before MPP^+^ treatment to confirm this hypothesis. BDS-II treatment protected SH-SY5Y cells against MPP^+^-induced neural cell death by inhibiting apoptosis. BDS-II treatment blocked mitochondrial cytochrome c release during MPP^+^ treatment. Several reports demonstrate K^+^ concentrations are an important factor in apoptosis^44^. Specifically, Kv2.1 is one of the most important potassium channels that are involved in apoptosis, and its functional role has been investigated in greater detail compared with other oxidation-sensitive channels^2, 45, 46^. Kv2.1, which is abundantly expressed in the cortex and hippocampus, is oxidized in the brains of aging mice, and oxidized Kv2.1 channels, which form oligomers through disulfide bonds involving Cys-73, accumulate in the plasma membrane as a consequence of defective endocytosis^47, 48^. The membrane accumulation of Kv2.1 oligomers disrupts planar lipid raft integrity and causes apoptosis by activating the c-Src/JNK signalling pathway^48^. Other Kv channels, such as Kv1.1, Kv1.3, and Kv4.2, are involved in neuronal apoptosis^49, 50^, and our data demonstrate that Kv3.4 is also involved in the neuronal apoptotic pathway.

Oxidative stress has been detected in a variety of neurodegenerative diseases, such as Alzheimer’s disease and Parkinson’s disease, and increasing evidence suggests that oxidative stress plays a major role in these diseases^51–53^. Several Kv channels, such as Kv2.1, Kv3.4, and Kv4.2, are thought to be connected to Alzheimer’s disease. Therefore, Kv channels, including Kv3.4, are expected to be novel therapeutic targets in Parkinson’s disease^14, 54, 55^. Sesti *et al*. (2010) suggested that the oxidation of K^+^ channels by ROS might be a leading cause of neurodegenerative diseases, including Alzheimer’s and Parkinson’s disease. They demonstrated that highly elevated ROS levels are found in the aging brain and in many neurodegenerative diseases and therefore, oxidative modification of K^+^ channels might be a general principle underlying aging and neurodegeneration^56^. Kv3.4 has been suggested as a new therapeutic target for major neurodegenerative diseases, and the mechanisms of Kv3.4 related to these diseases have been investigated^14, 57^. However, the relationship between the oxidative modification of Kv3.4 and neurodegenerative diseases remains unknown. We suggest that Kv3.4 may play important roles in neurodegenerative disease as an oxidation-sensitive channel.

Taken together, Kv3.4 has an effect on the death of SH-SY5Y cells as an oxidation-sensitive channel. Kv3.4 is involved in the MPP^+^-induced apoptotic pathway through, for example, cytochrome c release and MMP regulation. In addition, mitochondrial Kv3.4 may be important for such phenomena, and HIF-1α is a key regulator of Kv3.4. These data imply the pivotal function of Kv3.4 in the neuroblast cell as an oxidation-sensitive Kv channel, and further studies of the relationship between Kv3.4 and oxidative stress would provide a new therapeutic paradigm for oxidative stress-related diseases.

## Materials and Methods

### Cell culture

SH-SY5Y cells were cultured in a minimum essential media (MEM) (Welgene, Daegu, Korea) containing NaHCO_3_ supplemented with 10% fetal bovine serum (FBS) and 1% antibiotic-antimycotic solution (Sigma, St, Louis, MO) at 37% incubation with 5% CO_2_. When the cells grew sufficiently in a T75 flask (SPL Life Sciences, Gyeonggi-do, Korea), they were subcultured in 6-well plates or 100 mm culture dishes (SPL).

### Reverse transcription-polymerase chain reaction (RT-PCR)

Hybrid-R^TM^ (GeneAll, Seoul, Korea) was used to isolate the total RNA according to the manufacturer’s instructions. The cDNA was synthesized using 1 ug of isolated RNA with random hexamers and M-MLV (Promega, Madison, WI). The PCR reaction was performed with 2 ul of cDNA, 1 × GoTaq^®^ green master mix (Promega), and specific target primers in the following reaction conditions: initial denaturation at 94 °C for 5 min, 35 cycles of cycling process (94 °C for 40 s, the each of annealing temperatures (Kv3.3: 60 °C, Kv3.4: 65 °C) for 40 s, 72 °C for 1 min, and an extension at 72 °C for 1 min), and a final extension at 72 °C for 7 min. The PCR products were loaded on 1.6% agarose gel for electrophoresis and analyzed with an ABI Prism 3730 XL DNA Analyzer (Applied Biosystems, Foster City, CA) to confirm the channel mRNA expression in the SH-SY5Y cells.

### Western blot assay

SH-SY5Y cells were lysed using a radioimmunoprecipitation assay (RIPA) buffer (Sigma), and the total protein concentration was measured with a Bicinchoninic acid (BCA) protein assay kit (Pierce, Rockford, IL). The quantified protein was loaded on a 10% Sodium Dodecyl Sulfate Polyacrylamide Gel Electrophoresis (SDS-PAGE) and then transferred to nitrocellulose membranes (Whatman, Maidstone, Kent). Blocking was performed using 1x TBS-Tween 20 containing 5% nonfat milk (Difco, Franklin Lakes, NJ), and protein-transferred membranes were then probed overnight with target protein antibodies such as HIF-1α, Kv3.3, Kv3.4 (Abcam, Cambridge, MA), β-actin, COX-4, cytochrome c, or NDFIP1 (Santa Cruz Biotechnology, Finnell St., Dallas, TX). Membranes that were probed by primary antibodies were incubated with horseradish peroxidase–conjugated goat, anti-rabbit or anti-mouse secondary antibody (GenDEPOT, Barker, TX) for 1 hour and visualized using a WesternBright™ Quantum™ (Advansta, Menlo Park, CA).

### Real-time RT-PCR

Real-time RT PCR was performed using an Applied Biosystems StepOnePlus™ Real-Time PCR System (Applied Biosystems, Foster City, CA). Each of the primer efficiencies of the specific target primer was tested using a Glyceraldehyde 3-phosphate dehydrogenase (GAPDH) (a house keeping gene) primer as a reference. The standard curve for the efficiency test was obtained from 2- or 10-fold diluted cDNAs. The real-time RT-PCR reaction was performed with 2 μl of cDNA, 1x SYBR Green Master Mix (Applied Biosystems, Foster City, CA), and 0.2 μM forward and reverse primers (Kv3.3 forward: 5′-CCTTCCTGACCTACGTGGAG-3′, reverse: 5′-CGAGATAGAAGGGCAGGATG-3′, Kv3.4 forward: 5′-AATATCCCAGGGTGGTGACA-3′, reverse: 5′-GGTCTTCAAAGCTCCAGTGC-3′) using the following reaction condition: initial step 95 °C 30 s, then 40 cycles of cycling processes (95 °C for 5 s, and 60 °C for 45 s). The single product synthesized by the paired primers was analyzed with the use of a dissociation curve.

### Patch clamp recordings

The prepared SH-SY5Y cells for patch clamp recordings were incubated for 24 hours in 12-well plates containing 12 mm coverslips (SPL) with 2 ml of MEM medium to allow the cells to attach to the coverslips. The cell-attached coverslips were transferred to the chamber and were visualized through differential interference contrast video microscopy (Olympus, Tokyo, Japan). Patch pipettes were pulled from the borosilicate glass capillaries (1.7 mm diameter; 0.5 mm wall thickness) (World Precision Instruments, Sarasota, FL), resulting in a seal resistance ranging from 5 to 7 MΩ. The internal pipette solution (in mM concentration) consisted of 150 KCl, 1 MgCl_2_, 10 HEPES, 5 EGTA, and 2 Mg-ATP (pH 7.2 adjustment with KOH) and the bath solution (in mM concentration) containing 143 NaCl, 5.4 KCl, 0.5 MgCl_2_, 1.8 CaCl_2_, 0.5 NaH_2_PO_4_, 10 glucose, and 5 HEPES (pH 7.4 adjustment with NaOH) were used for recordings. The Kv channel currents were recorded in the whole cell configuration using an Axoclamp 2B amplifier (Axon Instruments, Foster City, CA). Electric signal filtering was performed at 1 kHz and digitized at 10 kHz with an analog–digital converter (Digidata 1320 A, Axon Instruments) and pClamp software (Version 9.0, Axon Instruments). The voltage-clamp mode was performed in the following protocol: cells were exposed to the hyperpolarizing pulse of −90 mV for 320 ms, and the membrane currents were activated by depolarizing pulses of 400 ms from a holding potential of −80 mV to test potential in the range of −70 to 40 mV in 10 mV increments. Membrane potential was recorded in the current-clamp mode.

### Cell viability test

Cell viability after 1 mM MPP^+^ treatment was analyzed by an MTT assay (Sigma). SH-SY5Y cells seeded in a 6-well plate were washed with Dulbecco’s phosphate-buffered saline (DPBS) and incubated with MTT solution for 4 hours. After the incubation, centrifuge the cells suspended in MTT solution to pellet the cells; SH-SY5Y cells are easily suspended, still alive, and, therefore, we applied the modified manufacturer’s protocol for non-adherent cells. The cell pellet was dissolved with dimethyl sulfoxide (DMSO), and 100 ul of the solution was then transferred to a new 96-well plate for measuring the absorbance at 570 nm. Apart from the MTT assay, the cell viability of 6-well plate–seeded cells was visualized with Hemacolor® rapid staining (Millipore, Billerica, MA) according to the manufacturer’s instructions. The cells were incubated for 30 seconds with each of the three Hemacolor® rapid staining solutions.

### Mitochondria isolation

Mitochondria were isolated from the SH-SY5Y cells using a mitochondria isolation kit (Life Technologies, Van Allen Way Carlsbad, CA). The isolation was performed following the manufacturer’s instructions, and a reagent-based method was used to isolate mitochondria. Cytosol and mitochondrial fractions were used for the western blot assay right after the isolation. The isolated mitochondrial fraction was confirmed with a COX-4 protein expression level as a reference.

### Mitochondrial membrane potential (MMP) measurement

Membrane potentials of mitochondria were measured with a JC-1 Mitochondrial Membrane Potential Assay Kit (Abcam) according to the manufacturer’s instructions. SH-SY5Y cells (1 × 10^6^) were seeded in a 35 mm confocal dish (SPL) and incubated for 24 hours to allow them to attach to the bottom of the dish. After washing the cells with DPBS, the cells were incubated with JC-1 solution for 10 minutes at 37 °C in the dark. The cells were washed again with DPBS and were treated with 1 mM of MPP^+^ for 4 hours, and 100 nM of BDS-II was pretreated 10 minutes before the MPP^+^ treatment if needed. MMP was analyzed using a confocal microscope at 535 ± 17.5 nm excitation for aggregated JC-1 only or 475 ± 20 nm excitation for both the aggregated and monomer forms of JC-1. The emission wavelength was 590 ± 17.5 nm.

FCCP (carbonyl cyanide 4-[trifluoromethoxy] phenylhydrazone) is an ionophore uncoupler of oxidative phosphorylation, and FCCP-treated cells were presented as an MMP depolarization positive control.

### Transfection of small interference RNA (siRNA)

Cells were transfected with HIF-1α or NDFIP1 siRNA purchased from Santa Cruz Biotechnology and Lipofectamine™ 2000 reagent (Invitrogen, Carlsbad, CA, USA) following the manufacturer’s instructions for adhesion cells. Control siRNA purchased from Santa Cruz Biotechnology was used for the control siRNA group. The SH-SY5Y cells (2 × 10^5^) were plated in 6-well plates in MEM (Welgene, Daegu, Korea) containing 10% FBS without antibiotics 24 hours before the transfection step. Adhered cells were transfected with siRNA and transfection reagent using MEM containing little FBS and no antibiotics for 5 hours. After 5 hours of transfection, fresh MEM containing 10% FBS and 1% antibiotics were given to the cells and the cells were incubated for 2 days. If CoCl_2_ treatment was used to induce HIF-1α accumulation, 100 µM of CoCl_2_ was added to the media 6 hours before the end of the transfection.

### Immunocytochemistry

The mitochondria of SH-SY5Y cells were probed with a 250 nM MitoTracker^®^ Deep Red FM (Life Technologies) during 30 minutes of incubation. After washing the cells twice with DPBS, the cells were treated with 4% paraformaldehyde for 20 minutes for fixation. The cells were washed again with DPBS and permeabilized using 0.3% Triton^tm^ X-100 (Sigma) for 5 minutes at room temperature. DPBS-washed cells were incubated with 5% donkey serum solution (Sigma) for 60 minutes at room temperature and then incubated with anti-Kv3.4 (Abcam) at 4 °C overnight. DPBS-rinsed cells were treated with 5% donkey serum solution containing Alexa Fluor® 488 dye (Life Technologies) for 60 minutes. After washing, the cells were mounted using VECTASHIELD Antifade Mounting Medium with DAPI (Vector Laboratories, Burlingame, CA). We assessed five different images in each group (the control and CoCl_2_ treatment groups) to avoid cherry-picking.

### ROS detection

ROS was analyzed using 2′,7′-dichlorodihydrofluorescein diacetate (2′,7′-dichlorofluorescin diacetate; H2DCFDA) (Invitrogen). SH-SY5Y cells were incubated with a medium containing 100 µM of CoCl_2_ or 1 mM of MPP^+^ until the indicated time points (0 to 6 hours). The cells were washed twice with DPBS and then incubated with 37 °C DPBS containing 5 µM of H2DCFDA for 30 minutes. Finally, the cells were incubated with a fresh medium for 15 minutes for recovery. Fluorescence images were taken using an Axio Scope (Carl Zeiss, Hallbergmoos, Germany) and digitally recorded with a cooled charge-coupled device (CCD) camera named Micromax Kodak1317 (Princeton instruments, AZ, USA). The images were analyzed using Metamorph version 6.3r2 (Molecular Devices Corporation, PA, USA).

### Statistical analysis

All data are shown as means ± standard error (SE). The Student *t*-test or One-way ANOVA was used to analyze the data and Tukey’s test was used as a post hoc test (GraphPad Prism version 5.0).

### Data availability

All datasets generated or analyzed during this study are included in this published articles and its supplementary information files.

## Electronic supplementary material

Supplementary Figure 1
