# Supplementary material for: Kv3.4 is modulated by HIF-1α to protect SH-SY5Y cells against oxidative stress-induced neural cell death
Source: Sci Rep. 2017 May 18;7:2075. doi: 10.1038/s41598-017-02129-w (PMC5437029; doi:10.1038/s41598-017-02129-w)
Supplement: Supplementary file 1 — Supplementary Figure 1 [file 41598_2017_2129_MOESM1_ESM.pdf]

Kv3.4 is modulated by HIF-1α to protect SH-SY5Y cells against oxidative stress-induced neural cell death

Min Seok Song, Pan Dong Ryu, So Yeong Lee*

Laboratory of Veterinary Pharmacology, College of Veterinary Medicine and Research Institute for Veterinary Science, Seoul National University, Seoul, 08826, Korea.

Corresponding Author:

So Yeong Lee, DVM, PhD

Laboratory of Veterinary Pharmacology

College of Veterinary Medicine, Seoul National University,

1 Gwanak-ro, Gwanak-gu, Seoul, 08826, Korea

Tel: + 82-2-880-1283

Fax: +82-2-879-0378

E-mail: leeso@snu.ac.kr

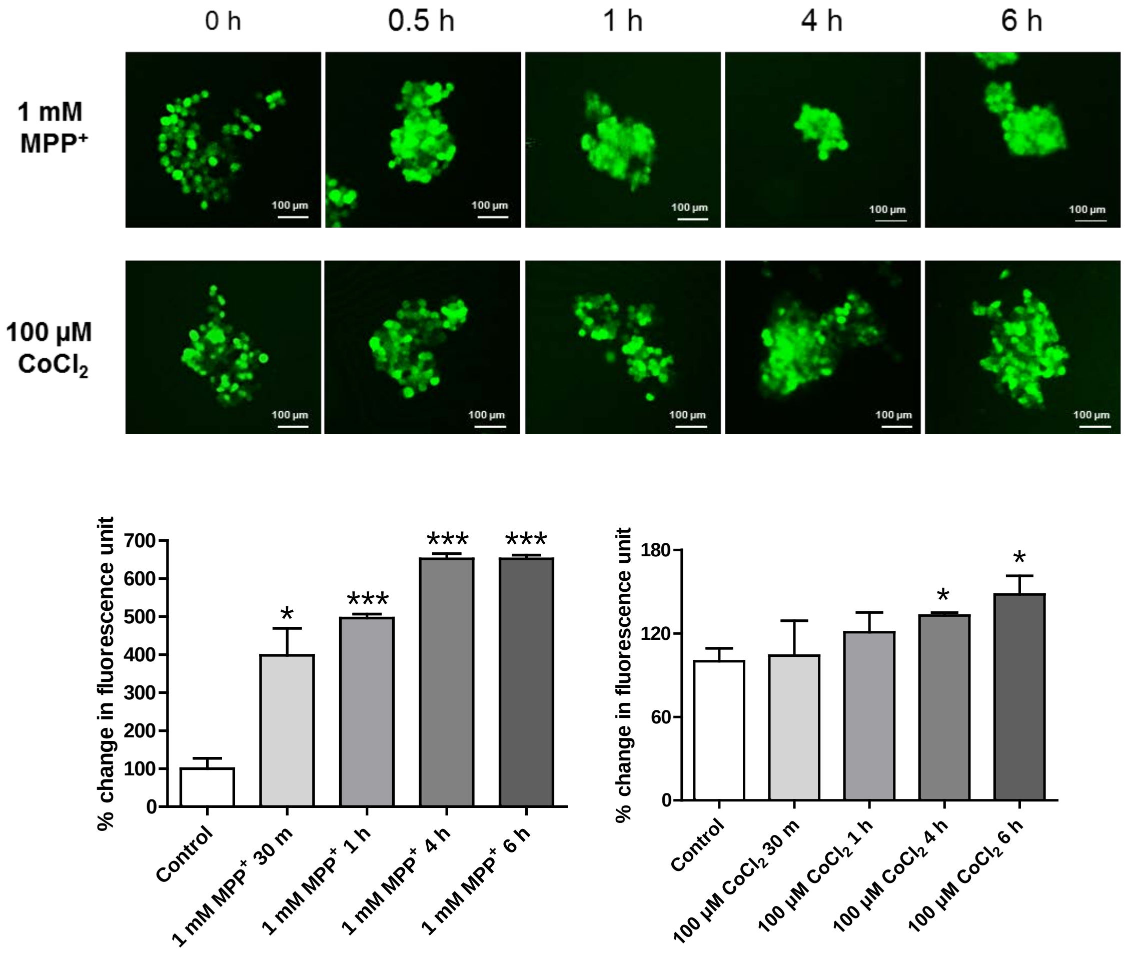

Supplementary Figure 1. MPP^+- or $CoCl_2$-induced oxidative stress measurement in SH-SY5Y cells Oxidative stress induced by MPP^+ or $CoCl_2$ treatment was measured in SH-SY5Y cells using the DCFH-DA method. MPP^+ effectively induced a large amount of oxidative stress after 30 min treatment, and the generated oxidative stress was saturated after 4 h treatment. On the other hand, $CoCl_2$ significantly induced oxidative stress after 4 hours of treatment. All experiments were performed in triplicate; representative images are shown.
